# The implementation of quality management systems in hospitals: a comparison between three countries

**DOI:** 10.1186/1472-6963-6-50

**Published:** 2006-04-11

**Authors:** C Wagner, L Gulácsi, E Takacs, M Outinen

**Affiliations:** 1 Nivel, Netherlands institute for Health Services Research, The Netherlands; 2 Department of Public Policy and Management, Budapest University of Economic Sciences and Public Administration, Hungary; 3 National Health Insurance Fund Administration, Hungary; 4 National Research and Development Centre for Welfare and Health STAKES, Finland

## Abstract

**Background:**

Is the implementation of Quality Management (QM) in health care proceeding satisfactorily and can national health care policies influence the implementation process? Policymakers and researchers in a country need to know the answer to this question. Cross country comparisons can reveal whether sufficient progress is being made and how this can be stimulated.

The objective of the study was to investigate agreement and disparities in the implementation of QMS between The Netherlands, Hungary and Finland with respect to the evaluation model used and the national policy strategy of the three countries.

**Methods:**

The study has a cross sectional design, based on measurements in 2000. Empirical data about QM-activities in hospitals were gathered by a self-administered questionnaire. The questionnaires were answered by the directors of the hospitals or the quality coordinators. The analyses are based on data from 101 hospitals in the Netherlands, 116 hospitals in Hungary and 59 hospitals in Finland.

Outcome measures are the developmental stage of the Quality Management System (QMS), the development within five focal areas, and distinct QM-activities which were listed in the questionnaire.

**Results:**

A mean of 22 QM-activities per hospital was found in the Netherlands and Finland versus 20 QM-activities in Hungarian hospitals. Only a small number of hospitals has already implemented a QMS (4% in The Netherlands,0% in Hungary and 3% in Finland). More hospitals in the Netherlands are concentrating on quality documents, whereas Finnish hospitals are concentrating on training in QM and guidelines. Cyclic quality improvement activities have been developed in the three countries, but in most hospitals the results were not used for improvements. All three countries pay hardly any attention to patient participation.

**Conclusion:**

The study demonstrates that the implementation of QM-activities can be measured at national level and that differences between countries can be assessed. The hypothesis that governmental legislation or financial reimbursement can stimulate the implementation of QM-activities, more than voluntary recommendations, could not be confirmed. However, the results show that specific obligations can stimulate the implementation of QM-activities more than general, framework legislation.

## Background

Since the 1990s, there is a general trend for stakeholders to put more pressure on hospitals for accountability, transparency and equity of access to health. The governments of various European countries have, therefore, stimulated the use of Quality Mangement systems (QMS) and external evaluation in healthcare. Former research has identified four principal models and national variants of external evaluation, e.g. medical speciality-driven visitation, traditional accreditation against explicit standards, European Quality Awards based on the model of the European Foundation of Quality Management (EFQM), and certification using ISO standards (ISO 9000 series)[1]. Although the evaluation models have common roots, their standards have been developed in response to national legislation, economics, culture and demand. The models share common principles and values, but have a different focus and are differently detailed. The perceived appropriateness of every model for hospitals is only one element influencing the prevalence of one approach over another [2,3]. Legislation also affects the use and development of external evaluation of hospitals. Some countries (e.g. Greece, Portugal and the UK) have no legal requirement for hospitals to meet specific organizational standards, whereas in other countries (e.g. Germany, France and Austria) governments have legislated some form of internal and/or external assessment of hospital services [4,5].

The objective of the study was to investigate agreement and disparities in the implementation of QMS between The Netherlands, Hungary and Finland with respect to the evaluation model used and the national policy strategy of the three countries.

**Figure 1:**
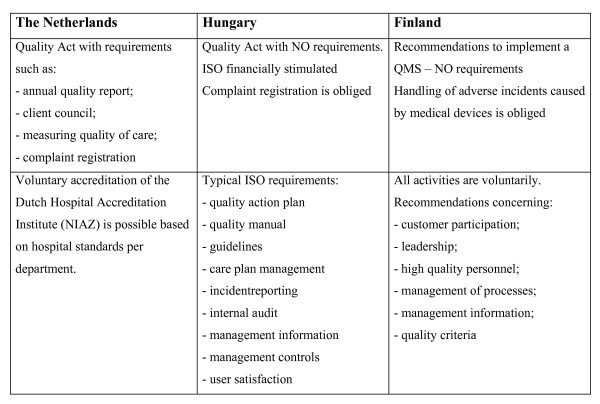
QM-activities obliged by law (The Netherlands), financial stimulation (Hungary), recommendations (Finland)

In this article, QMS is broadly defined as 'all the procedures explicitly designed to monitor, assess and improve the quality of care'. Examples are for example peer review, patient satisfaction surveys, complaints handling, audits, compiling a quality manual. The QM-activities that constitute a QMS were listed in a questionnaire. Disparities between the Netherlands, Hungary and Finland were expected due to the stimulating effects of the national quality policy and the Quality Acts and recommendations implemented. The most common evaluation model, the national policies and the country-specific quality legislation are explained below. Figure 1 shows the differences between the three countries.

## Dutch hospitals: quality policy and legislation

National quality requirements for Dutch hospitals are laid down in the 1996 Care Institutions Quality Act. This Act obliges all care organizations to set up a QMS to improve the quality of care. The QMS should reflect a cyclic process for monitoring, evaluating and, if necessary, improving the quality of care. The Act only provides a framework, no standards. It is up to hospitals to develop their own QMS and choose their own QM-activities and procedures; for example using protocols and guidelines, peer review, audits, benchmarking, satisfaction surveys. The Quality Act, however, requires that all care institutions provide clarity on the QM-activites and the quality of care by publishing an annual quality report which must be sent to the Ministry of Health, the Health Inspectorate and the regional patient/consumer organizations.

More and more hospitals are implementing the quality assurance standards of NIAZ (The Netherlands Institute for Accreditation of Hospitals). These standards contain requirements for the organization of a hospital. They describe what has to be regulated in a hospital in order to warrant that the quality of care delivered is not depending on individuals or left to chance. 35 Department-specific standards have been developed and one standard for the whole organization. Accreditation is a form of self-evaluation and peer review, and is aimed to enhance quality improvement. In addition, some hospital departments, such as laboratories, have an ISO certificate. There are no hospitals that have received an ISO certification for the whole organization. Hospitals are not no financially compensated for the implementation of QMS by health insurance funds or the Ministry of Health.

Since 1993, the Individual Health Care Professions Act governs the quality of professional practitioners. A statutory title protection and registration has been introduced for a number of professional practitioners, such as physicians, nurses, dentists and physical therapists. Continuous quality improvement by practitioners is required. The Act also contains a number of provisions to protect patients against incompetent treatment. Various procedures, such as administering injections, are restricted to a limited group of professionals. The medical specialists emphasise visitation, an external evaluation of peers, focusing on organisational aspects of the care process, and evidence-based guidelines [6,7].

## Hungarian hospitals: quality policy and legislation

In Hungary, the "Act CLIV of 1997 on Health" has a chapter entitled "Professional requirements for health care services" [8]. This chapter makes the operation of a QMS obligatory for every hospital. In addition, the implementation of ISO:9002 was financially stimulated.

The Act CLIV (1997) [9] sets out the objective of the QMS. According to paragraph 121 the aims of a QMS are:

a) to improve quality continuously, to explore and plan the process of service, the prevention of possible mistakes,

b) to reveal insufficiencies of delivery in time and to take and control necessary measures,

c) to explore the causes of insufficiencies, to decrease damages and expenses incurred by these,

d) to meet professional and quality requirements and to develop the institutions' own requirements.

The Act does not identify what kind of QM-activities should be performed in the daily practice, what kind of processes should be created, what kind of indicators should be set related to the structure, process or outcome [10].

### • Hospital Care Standards

In addition to the diffusion of ISO systems, health-specific systems are even more needed [11]. This gap was filled by Hospital Care Standards which are the adaptation of standards of the American Joint Commission. The manual for Hospital Care Standards was finally published in the Official Gazette of the Ministry of Health in 2001. The standards will be revised by the Ministry of Health every year according to change of law, experience and professional opinions. The main problem is that there is no clear accreditation because hospitals can only be certified by Hospital Care Standards. (Accreditation is only for labs in Hungarian health care. There is no Hungarian hospital accreditation body.)

### EFQM model of excellence

Approximately 10% of the hospitals carry out a self assessment based on the EFQM model. There is no health specific national quality award yet [12-14].

## Finnish hospitals: quality policy and legislation

In Finland, the municipalities (local authorities) are responsible for organizing and funding both primary and specialised care. Therefore, the responsibility is highly decentralised. There are approximately 450 municipalities, some of these are small with less than 2000 inhabitants. The municipalities have so far provided primary care in their own health centres, alone or together with neighbouring municipalities.

For the provision of specialised care Finland is divided into 21 hospital districts. Every municipality still has to belong to a hospital district for the provision of specialised care. The municipalities are the owners of the hospital districts through membership in the federations of the hospital districts.

The regulatory and monitoring role of the state has decreased along with the dismantling of norms and relative decrease in health care funding. The basic nature and operating framework for the health care services is laid down in law, but detailed questions of the scope, content, organization or quality of services are not included. Differences in health service provision from one municipality to another may occur.

No quality legislation has been passed in Finland, but national recommendations have been issued. The quality policy at national level is based on 'steering by information'. In stead of quality acts, four national recommendations have been issued in the years 1994–1999. In the most recent recommendation (1999) the recommendations are organized according to eight topics:

1 customer participation in QM;

2 leadership for the steering of quality;

3 personnel as a prerequisite for high quality;

4 QM for preventive as well other activities;

5 management of processes as a basis for QM;

6 information as a basis for the continuous enhancement of quality;

7 systematisation of QM;

8 detailed recommendations and quality criteria support quality management.

In addition to the quality management recommendations, there are several acts that can be considered as quality related acts, for example the Act Concerning Health Care Professionals (title protection, registration and disciplinary rules). For the legal protection of patients, Finland has issued the Status and Rights of the Patient Act in 1993. This Act includes a mandatory patient ombudsman and complaint handling, as well as use of care/treatment plans. The Patient Injury Act safeguards patients' interest in the event of malpractice. Handling of adverse incidents caused by medical devices is mandatory (Medical Devices Act).

Hospitals use several QM models and criteria for the development of their QMS. The Finnish Quality Award based on the EFQM-model is used in about 50 % of the hospitals as are ISO 9000 standards. But, only one hospital has an ISO-certificate for the whole hospital. The Finnish health care accreditation model is based on the King's Fund model (UK). It is used in about 20% of central, regional and private hospitals (15).

Most of the hospital clinical laboratories have received laboratory accreditation by FINAS and the majority of pathology departments a Pathology Quality Mark [15]. External audits became mandatory in radiology units in 2000, based on the Radiation Act.

## Hypothesis

The main underlying assumption in this article is that, in general, the three countries develop the same QM-activities, but, (expected) governmental legislation or financial stimulation can stimulate the use of QM-activities better than recommendations alone. Therefore, it was hypothesized that hospitals in a country with governmental legislation have a more developed QMS and develop more QM-activities. The second expectation is that financial stimulation is more effective than legislation only.

## Methods

The data for this study were collected in the winter of 1999/2000. A questionnaire was sent to all hospitals in the Netherlands, Hungary and Finland (a population survey). The addressees of the questionnaires were the managing directors of the hospitals, but, has been filled in most of the hospitals together with the quality manager. The questionnaire measured the extent of the implementation of QM-activities.

The questionnaire was developed in the Netherlands in 1995 and further improved in 1999. The reliability and validity of the questionnaire were tested in 1995 and are described elsewhere (16). The questionnaire had a closed, Likert-type format with two to four ordinally scaled response options per item.

A non-response analysis was conducted by telephone in 2000. The non-respondents were asked three questions: does the hospital have a quality manager, a quality policy and a guideline for patient information. It appeared that the non-respondents reported fewer QM-activities compared to the respondents. Therefore, a slightly positive bias in the results cannot be excluded.

### Data collection

**Figure 2:**
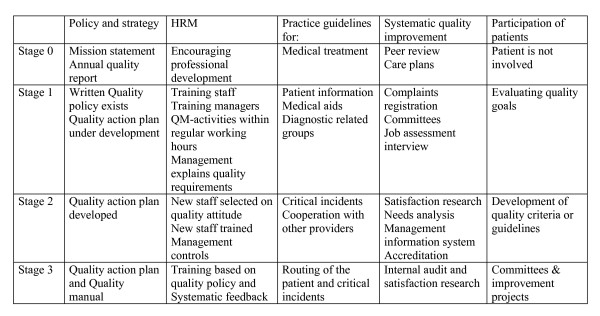
QM-activities divided into five focal areas and four developmental stages

The data collected concerned firstly the extent of QM-activities. The questionnaire listed various QM-activities (see figure 2). These items were empirically clustered into five focal areas, with the reliability coefficients given below.

## Analyses

For the description of QM-activities, percentages, means and ranges were used. For the analysis of the development of the QMS, the QM-activities were grouped into five focal areas. Per focal area and for the QMS as a whole, a score was computed. The reliability coefficients were:

1) the availability of quality policy documents (Cronbach's alpha .78)

2) human resources management (Cronbach's alpha .76)

3) using guidelines (Cronbach's alpha .71)

4) patient participation in QM (Cronbach's alpha .86).

5) quality improvement activities (Cronbach's alpha .80)

For the items in the first four focal areas, we used the affirmative answers (yes, this QM-activity is present in the organization). In the fifth focal areas, the items had three response options as follows: The quality improvement procedure: 1) is not present 2) is present, but not entirely operational 3) is present and operational. Operational was defined as meaning that the information obtained from peer review, audits or satisfaction surveys for example, is systematically used to make improvements. For the purposes of this article we combined the responses options 2 (present) and 3 (present and operational).

All missing values were recorded as zero, assuming that missing implied that the QM-activity was 'not present' in the organization. Hospitals with more than 5 missing activities were left out. Differences between countries were described if the differences were greater than 10%.

## Results

### Response

A total of 366 questionnaires was sent to hospitals in the Netherlands (N = 149), Hungary (N = 134) and Finland (N = 83). All hospitals were approached, including university hospitals, county hospitals and regional hospitals. 278 Questionnaires were returned. The overall response was 76%. The response rate in the Netherlands was 68% (N = 101), in Hungary 94% (N = 116) and in Finland 71% (N = 59).

### QM-activities

Table 1 gives an overview of the 38 QM-activities investigated and shows the percentage of hospitals in The Netherlands, Hungary and Finland performing these activities. The QM-activities are clustered into the focal areas: quality policy documents, human resources management, guidelines, QI-activities and patient participation.

#### Quality policy documents

Compared to Finland and Hungary, more hospitals in The Netherlands report that they have drawn up quality policy documents such as a mission statement (91%) and an annual quality report (97%). Nearly every hospital in the Netherlands publish an annual quality report, which is required by law. In Hungary and Finland 10% publishes such a report. In Hungary, more hospitals have written a quality manual (47%), but few have developed a written quality policy (37%). In general, less Finnish hospitals are concentrating on quality policy documents.

#### Human resources management

Overall, most attention has been spent on QM-training for professional staff and managers, and less to the training of new staff. The latter is important for the continuity of QM-activities. In Finland, more than 85% of the professionals and managers are trained in quality management. Compared to the Netherlands, fewer managers in Finland explain quality requirements to their staff (51% vs. 65%). In Hungary, more managers, i.e. 52% compared to 30% and 34%, control the compliance of staff with existing quality procedures. In the Netherlands, less attention is paid to new staff, i.e. 12% vs 24% and 29%. Minor differences exist with regard to training based on the quality policy.

#### Guidelines

More guidelines are reported in the Netherlands and in Finland compared with Hungary, e.g. patient information, critical incidents, diagnostic related groups and the cooperation with other health care providers. In Finland, most hospitals have guidelines for patient information and medical aids. In the three countries, most hospitals have guidelines for medical treatment. Less common are guidelines for the routing of the patient trough the hospital and for critical incidents.

#### Quality improvement(QI)- activities

It must be noted that the figures in the table represent the hospitals that apply the QI-activities, as well as the hospitals that use the results of the QI-activities for improvements of the care process.

In the Netherlands, more hospitals use mono-disciplinary peer review and fewer hospitals use need surveys among users and referrers. In Hungary, hospitals use more often internal audits, a management information system and need surveys among users. Finnish hospitals use more often satisfaction surveys among users and staff, and less often the complaint registration. Minor differences exist in the use of incident and infection committees, job assessment interviews, and user satisfaction surveys.

#### Patient participation

Minor differences exist with regard to patient participation between the three countries, except for the development of quality criteria. Nearly 40% of the hospitals in the Netherlands and Hungary invite patients for the development of quality criteria, whilst in Finnish hospitals this is only 14%. In general, the table shows that few hospitals invite patients to evaluate quality goals, participate in committees and improvement projects, or in the development of guidelines. Only in the Netherlands hospitals are obliged to have a client council and to discuss important topics with regard to the quality of care. In practice 63% of the Dutch hospitals have a client council (not in table 1).

The average number of QM-activities that have been developed in the three countries is 22 in the Netherlands and Finland, and 20 in Hungary.

### Country profile

**Figure 3:**
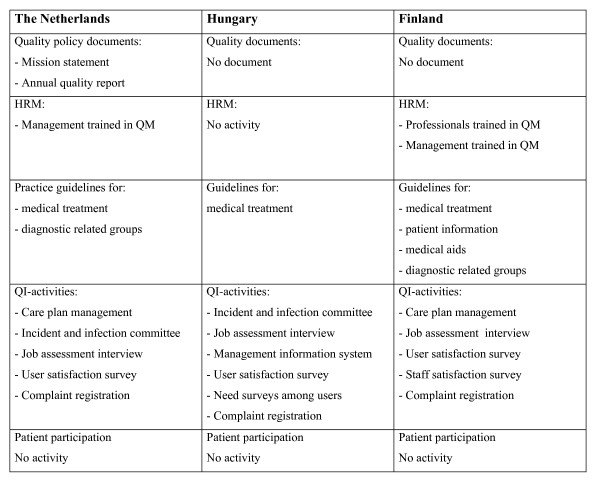
Strong points: QM-activities that 75% of the hospitals of a country has developed

Figure 3 gives an overview of the QM-activities that 75% of the hospitals of a country have developed. This figure, therefore, shows the strong points in the QMS of the hospitals. In the Netherlands, 75% of the hospitals have developed at least one QM-activity in the focal areas Quality policy, human resources management, guidelines, and cyclic QI-activities. In Hungary, the hospitals are concentrating more on the focal area QI-activities. Finnish hospitals are concentrating on training (HRM), guidelines for professionals and QI-activities. None of the three countries is concentrating on patient participation.

The weak points (<25%) in the Netherlands are the quality manual, feedback about results, training new staff and patient participation. Weak points for Hungary are the training of new staff, multidisciplinary peer review and patient participation. For Finland the weak points are the quality manual and patient participation.

### Developmental stage of the quality management systems

To determine the developmental stage of the QMS, the QM-activities within each focal area have been divided by national quality experts into four developmental stages, e.g. orientation and awareness (stage 0), preparation (stage 1), experimentation and implementation (stage 2), and integration into normal business operations (stage 3) (Figure 2). At stage zero, there are no systematic activities for quality assurance and improvement of health care processes. Some disciplines monitor their own quality through peer review and the use of standards for specific treatments. The management has started describing the mission, vision and products of the hospital. In this stage, the professionals are mainly responsible for quality assurance. At stage 1, hospitals create the conditions necessary for systematic quality assurance and improvement. At stage 2, hospitals develop different kinds of QM-activities and improvement projects. The purpose is to cross the boundaries of separate disciplines using the quality improvement cycle. At stage 3, the hospital reaches the stage of integration and establishment. Quality management is no longer an experimental activity, but is integrated into normal business operations. The results of QM-activities in one focal area will be used for changes and improvements in other focal areas. Therefore, it is necessary that hospitals develop activities simultaneously on more than one focal area.

Table 2 shows the developmental stage of the QMS for each focal area. Dutch hospitals are further with the participation of patients and one third of the hospitals has reached stage 3 for the focal area "Guidelines". Nearly one third of the Hungarian hospitals is in stage 3 for the focal area "Quality policy documents" and "QI-activities". Like the Dutch hospitals, more than one third of the Finnish hospitals has reached stage 3 for the focal area "Guidelines". A hospital can only reach a specific stage if it has developed the QM-activities of that stage. For example: a hospital can reach stage 3 of focal area "Quality policy documents" if it has developed a quality action plan and a quality manual (see Figure 2). Stage 3 hospitals have developed most of the QM-activities of the earlier stages.

Based on table 3, a small majority of Dutch hospitals (57%) has reached stage 2, 62% of the Hungarian hospitals have reached stage 1, and in Finland, half of the hospitals has reached stage 1 and half has reached stage 2.

Table 3 shows that there is no Hungarian hospital that has reached stage 3 for the overall QMS, despite the larger number of hospitals that has reached stage 3 of a specific focal area. These results seam to contradict each other. The explanation based on table 2 and table 3 is that a small group of Hungarian hospitals focuses on specific focal areas. They fulfil the requirements of a QMS within that area, but neglect the other areas. For the QMS as a whole, the five focal areas are equally important.

## Discussion and conclusion

The object of this study was to investigate agreement and disparities in the implementation of quality management systems (QMS) between the Netherlands, Hungary and Finland with respect to the quality model used and the national policy strategy of the three countries. The main assumption was that the three countries develop the same QM-activities, but that governmental legislation or financial stimulation are more effective in stimulating the use of QM-activities. Therefore, it was hypothesized that hospitals in a country with governmental legislation are further with the implementation of a QMS and the required QM-activities than hospitals in countries with recommendations. Another assumption was that financial stimulation will be even more effective.

## Strengths and weaknesses of the study

The main strength of the study is the measurement instrument which was thoroughly tested in former research [16,17]. The instrument provides detailed information about QM-activities, independent of specific characteristics of sub-sectors and independent of the quality model used by an organization (ISO, EFQM or other models).

The study's weakness is inherent in investigations conducted by questionnaires. We measured whether or not a QM-activity was present in an organization. The questionnaire approach did not permit us to assess the effectiveness of these activities in serving the purpose of quality assurance. The occurrence of social desirability in the responses could not be excluded. To diminish this effect, the hospital could report the activities anonymously to the researchers and it received individual feedback on the own results compared to the results of all the other hospitals (The Netherlands). Furthermore, social desirability seems unlikely because no sanctions were imposed on non-compliance with the Act in The Netherlands and in Hungary.

## The comparison between the three countries

In general, the differences between the three countries in the implementation of QMS and QM-activities are not as great as one would expect because of the different history of the countries and health care systems. The average number of QM-activities that have been developed in the three countries is 22 in the Netherlands and Finland, and 20 in Hungary. One specific activity of the Netherlands, the client council, does not exist in Finland or Hungary. All other activities are to some extent present in all the three countries.

The assumption was that hospitals in countries with a legal requirement of QMS would be further with the implementation of QMS than hospitals in countries where the implementation of QMS is voluntary. Therefore, hospitals in the Netherlands and Hungary are expected to be further than hospitals in Finland. This is not the case for Hungary.

The results show that only a minority of hospitals (less than 5%) has developed a QMS (stage 3). There is no real difference between the Netherlands and Finland – most hospitals have implemented various QM-activities, but only 3% – 4% have implemented an integral QMS. On the other side, hospitals in Hungary are less far than hospitals in Finland. Despite the ISO-certification of 30% of the Hungarian hospitals, most Hungarian hospitals are still in the preparation stage, whereas most Finnish hospitals have started with the implementation of the QMS. This means that the hypothesis has to be rejected.

An explanation for the little influence of national quality policy and legislation could be the broad definition of QMS. In the Netherlands and in Hungary, the Quality Act is only a framework legislation that states that hospitals should have a QMS, but it does not give information on the QM-activities that are needed for an integral system. Furthermore, this means that any kind of control or inspection whether hospitals fulfil the requirements of the law, is difficult and no sanctions are taken in both countries.

In addition, the results show that only the QM-activities that are stated by name in the Dutch Quality Act are present in more than 90% of the hospitals, e.g. annual quality report, infection and incident committee, and complaint registration. It seems that only clearly stated QM-activities have been implemented by the majority of the hospitals.

In Hungary, the implementation of ISO:9002 was financially stimulated. In order to receive the money, the management of the hospital had to show which QM-activities the hospital had developed. Therefore, it can be expected that a majority of the Hungarian hospitals have developed the typical requirements and activities mentioned in the ISO:9002 standard (Figure 1). The results do not entirely confirm this expectation. Less than half of the hospitals have implemented the necessary quality policy documents.

Overall, the results show that there is a lack of patient participation in the Dutch, Hungarian and Finnish hospitals. The results indicate that a law or financial reimbursement have some influence on the implementation of QM-activities if they are specific enough. More general, notions about QMS or quality improvement depend on intrinsic motivation of health care providers and hospitals and a QMS will not be implemented by the majority of the hospitals. More pressure seems to be necessary. On the other hand, Finland has no legislation stating mandatory measures and despite this, hospitals have implemented QM-activities and developed QMS.

## Future

In line with the conclusions, the national quality policy in the Netherlands and in Hungary is changing and the pressure on hospitals is increasing. In the Netherlands, a new national quality programme has been launched to stimulate hospitals and the healthcare inspectorate is asking for performance indicators.

In Hungary, the Health Care Act was completed by "The guideline of Health Ministry about internal quality management system of health care providers and connected requirements" and published in the Official Gazette of the Ministry of Health in 2002. This guideline sets out recommendations concerning the following fields:

a) managerial decisions to lead and coordinate,

b) providing human resources,

c) providing and using up material and financial resources,

d) planning, operating, evaluating and developing service processes,

e) evaluating internal quality system.

This guideline has a specific manual for implementation and was developed on the basis of ISO 9000:2000 standards, the EFQM model and the Hungarian Hospital Care Standards (12).

## Competing interests

The author(s) declare that they have no competing interests.

## Authors' contributions

The authors contributed equally to this work

## Note

Table 1 Percentage hospitals with QM-activities in the Netherlands, Hungary and Finland

Table 2 Percentage hospitals in stage 2 and stage 3 of the five focal areas of a QMS

Table 3 Percentage hospitals in the various developmental stages of the implementation of a QMS

## Pre-publication history

The pre-publication history for this paper can be accessed here:



## Figures and Tables

**Table 1:** 

**QM-activities**	**Dutch hospitals N = 101**	**Hungarian hospitals N = 116**	**Finnish hospitals N = 59**
**Quality policy documents**			
Mission statement	91	73	68
Quality policy	56	37	56
Quality action plan	41	35	27
Annual quality report	97	43	25
Quality manual	10	47	10
			
**Human Resources Management**			
Feedback to staff about results	19	40	34
New staff selected on positive attitude	29	41	44
Professionals trained in QM	74	70	95
Management trained in QM	77	68	86
Training based on quality policy	39	41	34
QM-activities within regular working hours	88	73	93
New staff trained in QM	12	24	29
Management explains quality requirements	65	60	51
Management controls compliance with procedures	30	52	34
			
**Practice guidelines for ...**			
Medical treatment	96	87	80
Patient information	74	36	92
Medical aids	65	66	81
Critical incidents	56	35	48
Diagnostic related groups	85	62	88
The routing of the patient	46	43	56
Cooperation with other providers	67	26	59
			
**QI-activities**			
Monodisciplinary peer review	70	28	59
Multidisciplinary peer review	60	21	63
Care plan management	81	72	81
Incident and infection committees	98	96	96
Job assessment interviews	97	97	100
Internal audits	44	65	53
Accreditation/certification	54	53	48
Management information system	58	83	63
User satisfaction surveys	82	95	100
Staff satisfaction surveys	54	49	92
Need surveys among users	25	84	46
Need surveys among referrers	36	53	53
Complaints registrations	93	96	78
			
**Patient participation in ....**			
Evaluating quality goals	22	20	22
Development of quality criteria	39	38	14
Committees & improvement projects	28	20	20
Development of guidelines	18	18	19
Average number of QM-activities	22 (SD = 6) Range 11–38	20 (SD = 8) Range 5–39	22 (SD = 6) Range 9–36

**Table 2:** 

**Focal area**	**Dutch hospitals N = 101**		**Hungarian hospitals N = 116**		**Finnish hospitals N = 59**	
	**Stage 2**	**Stage 3**	**Stage 2**	**Stage 3**	**Stage 2**	**Stage 3**
**Quality policy documents**	80	9	52	32	83	3
**HRM**	57	11	39	28	44	22
**Practice guidelines**	49	32	24	22	32	39
**QI-activities**	21	7	3	32	20	17
**Participation of patients**	15	28	23	16	20	10

**Table 3:** 

**Developmental stage QMS**	**Dutch hospitals N = 101**	**Hungarian hospitals N = 116**	**Finnish hospitals N = 59**
Stage 0	-	8	-
Stage 1	37	62	46
Stage 2	57	30	51
Stage 3	4	-	3
Average	1.7	1.3	1.6
